# Initial Design of Culturally Informed Behavioral Intervention Technologies: Developing an mHealth Intervention for Young Sexual Minority Men With Generalized Anxiety Disorder and Major Depression

**DOI:** 10.2196/jmir.2826

**Published:** 2013-12-05

**Authors:** Michelle Nicole Burns, Enid Montague, David C Mohr

**Affiliations:** ^1^Department of Preventive Medicine, Center for Behavioral Intervention Technologies (CBITs)Northwestern University Feinberg School of MedicineChicago, ILUnited States; ^2^Department of MedicineNorthwestern University Feinberg School of MedicineChicago, ILUnited States

**Keywords:** mobile health, eHealth, cultural competency, minority health, male homosexuality, male adolescents, young adult, anxiety, depression

## Abstract

**Background:**

To our knowledge, there is no well-articulated process for the design of culturally informed behavioral intervention technologies.

**Objective:**

This paper describes the early stages of such a process, illustrated by the methodology for the ongoing development of a behavioral intervention technology targeting generalized anxiety disorder and major depression among young sexual minority men.

**Methods:**

We integrated instructional design for Internet behavioral intervention technologies with greater detail on information sources that can identify user needs in understudied populations, as well as advances in the understanding of technology-specific behavioral intervention technology dimensions that may need to be culturally tailored.

**Results:**

General psychological theory describing how to effect change in the clinical target is first integrated with theory describing potentially malleable factors that help explain the clinical problem within the population. Additional information sources are then used to (1) evaluate the theory, (2) identify population-specific factors that may affect users’ ability to relate to and benefit from the behavioral intervention technology, and (3) establish specific skills, attitudes, knowledge, etc, required to change malleable factors posited in the theory. User needs result from synthesis of this information. Product requirements are then generated through application of the user needs to specific behavioral intervention technology dimensions (eg, technology platform). We provide examples of considerations relevant to each stage of this process and how they were applied.

**Conclusions:**

This process can guide the initial design of other culturally informed behavioral intervention technologies. This first attempt to create a systematic design process can spur development of guidelines for design of behavioral intervention technologies aimed to reduce health disparities.

## Introduction

Behavioral intervention technologies are patient-facing information and communication technologies (eg, websites, virtual reality, text messages) designed to improve physical and mental health by targeting behaviors and cognitions [1]. Although behavioral intervention technologies may have the potential to improve access to behavioral health care for underserved populations, we know of no well-articulated process for culturally informed behavioral intervention technology design. We describe the early stages of such a process, using the methodology that is currently being used for the initial design of a behavioral intervention technology targeting generalized anxiety disorder (GAD) and major depressive disorder (MDD) in young sexual minority men.

At the outset, we decided this intervention would be technology-based due to barriers to traditional care. Although barriers to mental health services affect youth in general [2], sexual minority youth face additional barriers such as reluctance to disclose to providers their sexual orientation and related issues [3,4]. Further, many psychologists are not trained to meet their needs [5]. Many young sexual minority men (22%) are uninsured, and 12% indicate they have nowhere to present for health care or information [6].

## Methods

As there were no validated psychological intervention models for anxiety or depression in sexual minority youth [7], we required a systematic design approach. The instructional design (ID) process for Internet behavioral intervention technologies [8] informed our thinking regarding identification of the users and usage contexts, and establishment of learning needs and goals. However, the ID process for Internet behavioral intervention technologies did not detail sources of information that can help to understand the users. Such information is particularly critical when the target population is understudied, and we addressed this in our process. We also endeavored to more broadly establish user needs, which include learning needs but also address other population-specific needs.

Thus, our behavioral intervention technology design process outlines sources of information to better understand the user and their cultural context. This information is used to generate user needs, which are then translated into product requirements. Requirements are organized using advances in the understanding of behavioral intervention technologies dimensions (ie, technology platform, functionality, content, and user interface) that may require cultural tailoring [9].

**Figure 1:**
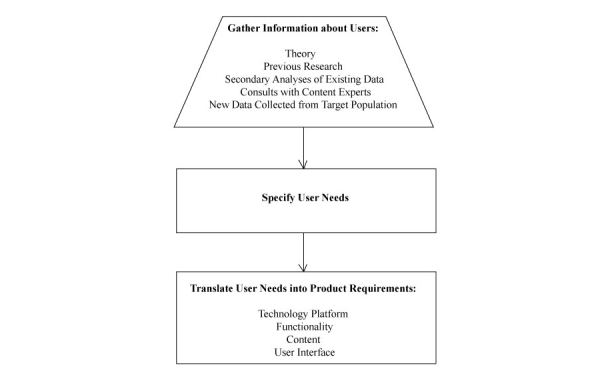
Process for the initial design of a behavioral intervention technology targeting generalized anxiety disorder and major depression among male sexual minority youth (rectangle=internal processes; trapezoid=process resulting in the input of information).

This paper focuses on the question of how to arrive at an initial conception of a behavioral intervention technology. While evaluation of the perceived usefulness, usability, and feasibility should occur throughout the design process [8], evaluation methods are beyond the scope of this paper. We suggest readers consult user-centered design [10] to choose evaluative methodologies for each phase of their design process. Each step of our initial design process is displayed in Figure 1, and an example is described in Table 1.

**Table 1:** Example of how a piece of information gathered about the users is used to specify a user need, which is then translated into a product requirement.

Information gathered about the users	User need	Product requirement
Minority sexual orientation is a relatively concealable, stigmatized characteristic that can pose safety risks if revealed to others.	Private access to the intervention.	A study-provided mobile phone will offer private access to the intervention, which will also be password-protected.

## Results

### Step 1: Gather Information About Users

#### Overview

We first defined characteristics of our users: (1) males who are attracted to other males, (2) aged 17-20, and (3) experiencing GAD and/or MDD. Although sexual orientation refers to multiple facets of sexuality including attraction, behavior, and identity (eg, self-identification as “gay”, etc), there is some evidence that attraction, unlike behavior and identity, is considered by adolescents to be critical in terms of defining one’s sexual orientation [11]. Thus, attraction to other males was the only defining characteristic in terms of the target population’s sexual orientation.

We next gathered information regarding ways that each of the defining user characteristics may influence behavioral intervention technology design, preferring any available information relating to their intersection. Our objectives were to establish (1) user needs relating to the “gap” between optimal behaviors, thoughts, and feelings versus current behaviors, thoughts, and feelings in this population, (2) knowledge, skills, attitudes, etc, needed to enable users to close this gap [8], and (3) user needs regarding behavioral intervention technology feasibility and acceptability.

We will now describe types of information that can be gathered to help answer these questions. We suggest first selecting theory, as theory helps establish the other types of required information. We next recommend consulting previous research to evaluate the evidence supporting the theory and identify user characteristics that could influence the way users relate to and engage with behavioral intervention technologies. A review of other previous research may also be necessary depending on the theory, and we will describe an example.

Other sources of information can augment theory and research findings. Consultation with content experts can be a rich source of clinical data throughout the information gathering and subsequent stages of this design process. In addition, secondary analysis of existing data and collection of new data from users are being employed to fill in gaps of the research literature.

#### Theory

ID for Internet behavioral intervention technologies suggests using theory to guide understanding of user needs; in our case, we required general theory describing how to bring about affective change [8]. We also needed to expand the conceptualization of theory to include population-specific theory. As young sexual minority men are at increased risk for anxiety and depressive symptoms [12,13], we required theory elucidating potentially malleable factors that explain this disparity.

##### General Theory of Affective Change

Internet interventions based on principles of cognitive behavioral therapy (CBT) for anxiety, depression, and stress have demonstrated efficacy when supported by brief provider contact [14]. Literature is growing that supports cognitive behavioral Internet interventions for adolescents with depressive and anxiety disorders [15]. Thus, the cognitive behavioral model was used as a framework for affective change. Due to high depression and anxiety comorbidity, shared mechanisms have been identified and used to create unified CBT protocols for adults and youth [16,17]. Unified cognitive behavioral theory describes putative maladaptive thoughts and behaviors common to individuals with depression and anxiety (ie, overestimation of the probability and consequences of negative outcomes, avoidant emotion regulation strategies) and thoughts and behaviors that are optimal in promoting affective change (ie, realistic cognitive appraisals and adaptive emotion regulation strategies) [16]. Thus, unified cognitive behavioral theory has outlined the differences, or “gaps”, between existing versus optimal thoughts and behaviors, as well as skills that can be taught to help users close these gaps. The cognitive behavioral model also has implications for the contexts in which users should interact with the intervention, as it requires that patients apply their learning during problematic situations encountered in daily life.

##### Theory of Mental Health Disparities Among the Users

###### Overview

Ample evidence supports the minority stress model, which implicates stressors generated by stigmatizing social contexts (eg, discrimination, victimization) in mental health disparities among sexual minority people [3,18,19]. Unlike some other stigmatized identities, sexual orientation may be concealable, and such concealment is also conceptualized as a form of minority stress [18]. However, disclosure of sexual orientation can also be a source of minority stress, as disclosure can place youth at risk for victimization [20,21].

**Figure 2:**
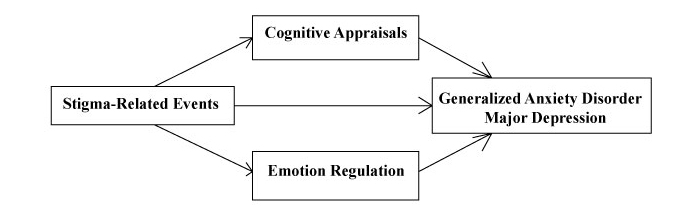
The population-specific theory of affective change used to guide the design of a behavioral intervention technology targeting generalized anxiety disorder and major depression among male sexual minority youth.

The psychological mediation framework [22] extended the minority stress model by proposing pathways between stigmatizing events and mental health. Many of these pathways (eg, appraisals of social support, rumination) are malleable according to unified CBT protocols, as they involve maladaptive cognitive appraisals and emotion regulation. Thus, we integrated unified CBT with the most clearly compatible aspects of the psychological mediation framework (see Figure 2) to derive a population-specific model of affective change to guide the behavioral intervention technology design.

#### Previous Research

We searched previous literature for evidence regarding the population-specific model of affective change. Prospective and experimental evidence suggests that emotion dysregulation does mediate the relationship between stigma-related events and psychological distress [22]. Regarding cognitive appraisals in our population-specific model of affective change, discrimination, victimization, and concealment may negatively influence cognitive appraisals of social support and thus increase depression and anxiety [22,23]. In partial support of this idea, lower perceived social support has been found to predict depression among gay youth [24]. The evidence for the population-specific model of affective change was limited, however, as most research with sexual minority people is cross-sectional [25], obscuring the ways minority stress processes interrupt healthy development [26]. Also, as very large samples are required to recruit representative sexual minority samples, convenience samples are often used that overrepresent white individuals and those who identify as gay [27,28].

As cognitive behavioral principles are designed for use in distressing situations, we also used previous research to identify contexts in which our users would likely encounter minority stress. Unlike many other stigmatized characteristics, families are unlikely to share the youth’s sexual orientation and may be hostile toward it [29]. Sexual minority youth are also more likely than heterosexual youth to be victimized at school and skip school due to safety concerns [3]. Adolescent males attracted to both males and females report lower academic achievement than their male heterosexual peers [30], and this may be due to hostile school climates [31]. Young sexual minority men can also lose friendships over their sexual orientation [32]. Thus, sexual minority youth can experience stigma-related events in the context of family, school, and friendships, all of which are key domains in the lives of many young people.

Previous research regarding acceptability and feasibility considerations was then examined. One of the first steps to developing a culturally inclusive design was gaining better understanding of how users may conceptualize their sexual identity. Little work has been done to develop design requirements for sexual minority people, so we began with examining the language young men use to describe their sexual orientation. In contrast to labels such as “gay” or “bisexual”, many adolescents appear to better understand their sexual orientation in terms of the sex(es) to whom they are sexually attracted [33]; indeed, some youth reject sexual orientation labels entirely [34]. For others, a wide variety of terms are used (eg, “pansexual”).

Finally, we sought research on the implications of user characteristics that are not related to sexual orientation for behavioral intervention technology feasibility and acceptability. First, a key developmental task of adolescence for both sexual minority and majority youth is to increase autonomy [3,35]. Second, metacognitive skills can be impaired by depression [36]. Third, due to mood-congruent recall [37], it may be difficult for users to remember how to implement new skills when they feel depressed or anxious.

#### Secondary Analyses of Existing Data

When designing behavioral intervention technologies for an understudied population, researchers may expect limitations in the literature regarding evidence for their model of population-specific change. Consequently, we are conducting a secondary analysis of data from an ongoing longitudinal cohort study of 451 racially diverse sexual minority men aged 16-20 (first reported by Mustanski et al [38]), recruited in Chicago using a sampling method that facilitated enrollment of young men unaffiliated with the gay and bisexual community. Respondent driven sampling [39] was used and modified to allow a higher percentage of initial recruits (ie, “seeds”), who were located through community outreach, school organizations, flyers posted in community locations frequented by LGB youth, and geosocial network applications (ie, Grindr and Jack’d). The seeds were then compensated for recruiting up to 3 other young sexual minority men into the study. New recruits, in turn, could also earn monetary incentives by recruiting up to 3 of their peers into the study.

Participants were given measures of minority stress, cognitive appraisals, and anxiety/depressive symptoms every 6 months. Ongoing, unpublished analyses are thus far highlighting the importance of appraisals of family support in predicting anxiety/depressive symptoms 6 months later. Although the inclusionary definition of sexual orientation differed in this longitudinal study from our defined target population, the analyses provided some support for the pathway between cognitive appraisals and anxiety/depression in our population-specific model of affective change. Had there been published trials of behavioral interventions for anxiety or depression with this population, we would have also requested to analyze such data to ascertain whether our model of affective change mapped onto mechanisms of action.

#### Consultation With Content Experts

To work toward culturally informed implementation of evidence-based principles, content experts should review the researchers’ understanding of gathered information. We have begun discussions with mental health professionals at a community organization serving sexual minority youth regarding our population-specific model of affective change, preliminary results of our secondary analysis, and their implications for intervention strategy. Further, the research team includes a psychologist and a physician with expertise on the health of male sexual minority youth, an adolescent psychologist with expertise in CBT for anxiety and depression, an expert in behavioral intervention technology development (DCM), and a software engineer. This team only begins to speak to the need to define “content experts” broadly, due to the multidisciplinary nature of behavioral intervention technology design.

#### New Data Collected From the Target Population

Information gathered thus far suggests the tentative learning goals are to increase perceived family support or help youth source positive social support from other individuals, and foster adaptive emotion regulation strategies. However, as young sexual minority men are understudied, the knowledge, skills, behaviors, and attitudes needed to meet these learning goals are unclear. A few qualitative studies have been conducted with samples that were not characterized on the basis of mental health, indicating sexual minority youth use a variety of strategies and resources to cope with minority stress (eg, [40]). However, it is difficult to evaluate the adaptive potential of these strategies without specifically examining youth who experience minority stressors without becoming distressed.

Accordingly, we have designed qualitative interviews to conduct with a subset of young sexual minority men participating in the aforementioned longitudinal study; we are inviting participants who have repeatedly (ie, at their two most recent assessments) evidenced nonclinical anxiety and depression symptom scores, despite repeatedly experiencing discrimination, to participate in the qualitative interviews. By understanding adaptation among youth who are resilient to minority stress, we will begin to understand how, specifically, male sexual minority youth can remain emotionally regulated and protect their perceptions of family support in the face of minority stress.

### Step 2: Specify User Needs

Due to the potentially concealable nature of sexual orientation, and the fact that its disclosure could result in discrimination or victimization, the paramount user need is private access to the behavioral intervention technology.

As stated previously, the behavioral intervention technology also needs to help youth improve appraisals of family support, or build or strengthen other social supports, and replace maladaptive with adaptive emotion regulation strategies. Subsumed user needs (eg, the skills, knowledge, and attitudes required to fulfill these broader learning needs) remain unknown, as we have not yet conducted the qualitative interviews regarding resilience to minority stress. Resources are often too limited to gather all desired information sources prior to applying for funding. Other researchers may also find it necessary to partially specify user needs and translate them into product requirements to create a thoughtful proposal and seek such funding. We thus conceptualize the information gathering phase as continuing to occur during the other phases of our process, enabling refinement of user needs and product requirements as new information becomes available.

The behavioral intervention technology must address minority stress driving higher rates of anxiety and depressive symptoms among the target population, and this is more likely if therapies actively engage youth in these stigmatizing social contexts [41]. As sexual minority youth may face stigma in their family, school, and social life, and CBT relies on use of new behaviors and ways of thinking during distressing situations, users need the ability to access the behavioral intervention technology during as much as of their daily life as possible. Users who report distress also need prompts to remind and guide them to use cognitive behavioral skills in the moment.

Due to educational disruptions or depression, some users may need the behavioral intervention technology to accommodate lower levels of literacy and metacognitive skills, respectively. Given their developmental stage, users also need the behavioral intervention technology to convey respect and support for their growing need for autonomy. Finally, the language of the behavioral intervention technology needs to be compatible with users’ understanding of their sexual orientation.

### Step 3: Translate User Needs Into Product Requirements

#### Overview

Within this phase, we have subsumed the recently published model of specific features of patient-facing behavioral intervention technologies that require cultural consideration, which comprise (1) technology platform, (2) functionality, (3) content, and (4) user interface [9]. We now describe several examples of how user needs inform each of these four areas.

#### Technology Platform

A mobile phone platform meets the need for ubiquitous technology resulting from the varying contexts in which minority stress takes place and the corresponding need for application of cognitive behavioral skills in the face of minority stress. Also, a purely Web-based intervention may not meet the user need for private access to the behavioral intervention technology. Youth may not have a point of access to the Web that is not monitored by others. Thus, for the duration of the intervention, users will be provided with a mobile phone to allow private access to the behavioral intervention technology. The behavioral intervention technology will also be password-protected to protect the youth’s data if the phone is lost or stolen.

#### Functionality

The extent and nature of interactivity, amount of user control, sequencing of information, and rate of delivery [42] should be selected based on user needs [8]. To reduce the metacognitive skill required, we are using short, scaffolded didactic information and frequent interactive experiences with corrective feedback [42]. Accordingly, each day the application will provide users with either a small chunk of information or a brief, interactive skills practice with clear linkage to previous material. Further, the mobile phone application will prompt users several times daily to report on their mood. The behavioral intervention technology will guide users reporting distress through tools that will scaffold them in enacting behaviors that are adaptive given their current discrete emotion(s) and intensity. For example, youth reporting moderate guilt will be routed to tools that target guilt by restructuring maladaptive cognitive appraisals, such as by focusing on how to learn from a mistake. In contrast, youth experiencing intense guilt would first be walked through distress tolerance exercises [43,44], such as focusing on the sensations of one’s feet touching the ground. Another example of feedback is that the phone application will offer visualization tools to show users how their emotions relate to their behaviors and contexts. For example, youth can see a graph showing their average level of sadness in each location in which they have reported their mood.

The intervention “functionality” also includes human coaching, which is commonly used to increase adherence to Internet behavioral intervention technologies [45]. As mentioned, the behavioral intervention technology depends on feedback [42], and there are some tasks (eg, completing a thought record) that may be too complex for the mobile phone application to provide meaningful feedback. The behavioral intervention technology will enable coaches to view users’ work, such that coaches can provide feedback for more complex tasks. In keeping with the goal for the behavioral intervention technology to foster frequent but short interactive experiences, coaching contacts will include a brief telephone call (5-10 minutes) and email each week. A coaching protocol (“TeleCoach”) [46] based in part on motivational interviewing [47] will be used to maintain engagement in treatment despite avoidance behaviors that may arise due to anxiety and depressive symptoms. Coaches will be clinicians with expertise in motivational interviewing.

#### Content

The need for autonomy among youth indicates that a collaborative, empowering tone is appropriate for the content. For example, the intervention will first involve personal goal-setting that will be used to frame the other intervention components. Also, the behavioral intervention technology content around loss of family support will stress the youth’s emerging independence and ability to obtain social support from other sources (eg, the idea of building a “family of choice” [3]).

Consistent with the many domains in which sexual minority youth can encounter minority stress, the content will also address minority stress occurring in a variety of contexts. Skills such as cognitive restructuring, problem solving, and finding alternatives to avoidant emotion regulation strategies are taught in part through the use of examples. Such examples include a young man encountering bullying at school, a youth who has lost a good friend after he disclosed his sexual orientation, and a young man trying to problem solve regarding how and when to disclose his sexual orientation to his parents. Examples will be enhanced by incorporation of young sexual minority men’s narratives collected during the qualitative interviews regarding resilience to minority stress.

#### User Interface

Users will be able to obtain the same information from audio voiceovers, illustrations, and videos as contained in the text, to accommodate possible educational disruptions due to school victimization [8]. The terminology used to describe sexual orientation will also be inclusive (eg, “attracted to men” versus “gay” or “bisexual”). When discussing sexual identification, a spectrum of possible sexual orientation labels will be acknowledged.

## Discussion

### Principal Results

Design of behavioral intervention technologies for underserved populations can be guided by general psychological theory describing how to effect change in the clinical target, integrated with theory describing potentially malleable factors that help explain incidence of the clinical problem among the population. Previous research, analysis of existing data, consultation with content experts from multiple disciplines, and collection of new data can then be used to (1) evaluate the guiding theory, (2) identify group-specific factors that are not learning needs, but may affect users’ ability to relate to and benefit from the behavioral intervention technology, and (3) establish specific skills, attitudes, knowledge, and behaviors required to change malleable factors posited in the theory. User needs result from synthesis of this information. Generation of product requirements is then guided by application of the user needs to choice of technology platform, functionality, content, and user interface.

### Limitations

Future work should extend this process model by articulating methods to evaluate each of its stages, as well as incorporating cultural intersectionality, the idea that multiple identities (eg, Latino, bisexual, male) are not additive and cannot be fully understood in isolation [48].

### Conclusions

This early design process is readily understandable to clinical scientists and thus can be translated to serve interventionists targeting other populations or clinical targets, as well as those interested in creating a model for the entire design process of culturally informed behavioral intervention technologies. The process of gathering information and defining user needs is also applicable to the development of interventions that do not involve technology. The model could also assist clinicians in culturally tailoring their interventions for patients from underserved populations, as well as to spur further discussions of guidelines and standards in the development of behavioral intervention technologies aimed to reduce health disparities.

## Abbreviations

CBT: cognitive behavioral therapy
GAD: generalized anxiety disorder
ID: instructional design
MDD: major depressive disorder
